# Patients’ motives and considerations on treatment decision-making for heavy menstrual bleeding: a qualitative study

**DOI:** 10.1186/s12905-024-03268-9

**Published:** 2024-08-02

**Authors:** T. J. Oderkerk, R. G. Singotani, L. Zuidema, E. J.E. van der Hijden, P. M.A.J. Geomini, M. Y. Bongers, M. H. Donker

**Affiliations:** 1 Department of Obstetrics and Gynecology, Máxima Medical Center, P.O. Box: 7777, Veldhoven, MB 5500 The Netherlands; 2 Department of Obstetrics and Gynecology MUMC+, Grow-School of Oncology and Reproduction Maastricht University, Maastricht, The Netherlands; 3 Department of Ethics, Governance, and Society, School of Business and Economics, VU University Amsterdam, Amsterdam, the Netherlands; 4 Department of Health Sciences, VU University Amsterdam, Amsterdam, the Netherlands

**Keywords:** Heavy menstrual bleeding, Qualitative study, Treatment decision-making

## Abstract

**Background:**

Several treatment modalities for heavy menstrual bleeding are available. However, many women report being unsatisfied in their search for an appropriate and effective treatment. The aim of this study is to gain insights in the experienced impact of heavy menstrual bleeding and the motives and considerations of women during the decision-making process for treating heavy menstrual bleeding.

**Methods:**

An interpretative qualitative study was performed, using in-depth interviews. In total, 14 semi-structured interviews were conducted with patients who consulted a physician for treatment of heavy menstrual bleeding. Participants were recruited via the Netherlands Patients Federation (*N* = 10) or via the outpatient clinic in the Máxima Medical Center (*N* = 4). The interviews were conducted by phone or online between February 2020 and March 2021. In the interviews three topics were addressed: (1) participant’s experience with heavy menstrual bleeding, (2) experience with patient journey of treatment decision-making and (3) elaborating on alternative treatments for heavy menstrual bleeding. A thematic analysis was conducted.

**Results:**

Fourteen participants aged between 30 and 59 years old were interviewed. Three main themes emerged; “Considerations in taking the (next) step to seek help”, “Various sources of information can contribute, confuse or frighten decision-making process” and “A physician’s understanding and a relationship of trust are needed to guide the decision-making process”.

**Conclusion:**

Our results show that women’s considerations and decision making strongly depend on the obtained information and experience, the relationship with the physician, the influence of the social environment, the pre-visit expectations/desires, the fear of treatment complications and uncertainty of the effect of the treatment. It is a physicians role to create a trusting and open atmosphere during consultation. Patient-centered communication is helpful to share knowledge, and gain insights into a patient’s hopes, fears and worries.

## Background

Heavy menstrual bleeding (HMB) has had many definitions over time, but is now defined as experiencing excessive menstrual bleeding that interferes with women’s physical, emotional, social and material quality of life. HMB affects roughly 30% of European women in reproductive age [1, 2]. Among women with HMB, only one in two seeks consultation from a physician, of which 70% receives treatment [2]. The most frequently reported reason to refrain from seeking help is a lack of awareness about the disease and its treatment options [2]. Additionally, HMB is often associated with social stigmas and personal beliefs about menstruation, which can affect women’s decisions to seek treatment [3]. However, there are several treatment options available for managing HMB. Usually, the first initial step in treating HMB is a medical treatment such as the combined oral contraceptive (COC), tranexamic acid or inserting insertion of progesterone via an intra-uterine system. The levonorgestrel releasing intra-uterine device (LNG-IUD) is a hormonal contraception that has been proven effective for treating HMB as it induces atrophy of the endometrial tissue [4, 5]. Endometrial ablation or resection (EA) is a viable option for women who do not wish to have children, or no longer intend to have children, as pregnancy after EA can cause serious obstetric complications [6]. This treatment aims to stop endometrial growth by ablating the endometrial tissue and superficial myometrium [7]. While a hysterectomy is typically considered the most effective treatment option, it is a major and definitive procedure for treating HMB.

Given the various treatment options for HMB, with each treatment having its own risks and benefits, women may not be aware of the numerous available options [8, 9]. Therefore, it is crucial to collaborate with women to explore different treatment options, respecting and prioritizing their preferences. A (shared) decision-making process involves collaboratively exploring the best available evidence with patients and supporting them in making informed decisions that align with their values [10]. Patient-centered communication can help to uncover and integrate patients’ wishes, feelings, illness beliefs, concerns, expectations, and preferences during consultation [11]. According to Zandstra et al. (2017) effective counseling and shared decision-making are essential to help patients navigate their options and make choices that align with their personal values and needs [8]. They specifically examined women’s preferences on the kind of (shared) decision-making. They concluded that information packages did not influence treatment choice, however structured interviews and computerized decision aids, which were integrated with patient preferences, were beneficial for treatment choice, reducing disagreement, and increasing patient satisfaction [8]. However, the reviewed studies did not elaborate on the specific motives and experiences in the decision-making process that led to the decision for a treatment choice. Therefore, we performed this interpretative qualitative study to gain insights in the experienced impact of HMB and the motives and considerations of women during the decision-making process regarding treatment options for HMB.

## Methods

### Study aim and design

To gain insights in the experienced impact of HMB and the motives and considerations of women during decision-making for different treatment options for HMB, an interpretative qualitative study was performed, using in-depth interviews.

### Participants

Participants were recruited using purposive sampling through the Netherlands Patients Federation (NPF) and Máxima Medical Center (MMC). A specific questionnaire was developed to recruit members of the NPF. The questionnaire contained information on the study aim and questions addressing the inclusion criteria (experience with HMB; received treatment for HMB in the previous five years). Initially, only women experiencing HMB and treated with an LNG-IUD or EA were included. Criteria were broadened after five interviews, including women treated with other treatments such as oral (hormonal) medication and hysterectomy. Additional participants who received any treatment for HMB were recruited via MMC and they received an information leaflet. All women that showed interest in the study, participated in an interview. Women who never consulted a physician for treatment of HMB were excluded.

### Data collection

Semi-structured, in-depth interviews were conducted in Dutch, between February 2020 and March 2021 and lasted approximately 30 to 90 min. The majority of interviews were held during the COVID-19 pandemic and were carried out online or by phone. All interviews were conducted by two female researchers (RGS and TJO). RGS was a researcher with a background of qualitative research; TJO is a researcher and medical doctor in a gynecology department. Fieldnotes were made during the interview. A topic list was used as a guide during the interviews (see Appendix A). The topic list was inspired by two models for treatment decision-making [10, 12, 13]. Questions were developed to reconstruct patient journeys and gain insights on the motives and considerations of participants during the decision-making process regarding treatment options. Topics that were discussed included: (1) participant’s experience with heavy menstrual bleeding, (2) experience with patient journey of treatment decision-making and (3) elaborating on alternative treatments for heavy menstrual bleeding. The topic list included questions such as “What was the most important goal you wanted to achieve when visiting your physician?”. The process of data collection and analysis was iterative, alternating between them. After five interviews, inclusion criteria were broadened and women who received any treatment for HMB were recruited. Additionally, after five interviews an option grid was introduced to discuss treatment options for HMB. This grid explained advantages and disadvantages of various treatment options for HMB offered in the Netherlands. The option grid was sent prior to the interview. After eight interviews, no new main themes emerged.

All interviews were audiotaped and transcribed verbatim. Data was collected until no new themes emerged.

### Data analysis

A descriptive thematic analysis was conducted using the revised six steps of thematic analysis as described by Braun & Clarke (2019) [14]. In the first phase, the researchers (TJO and RGS) familiarized themselves with the data by re-reading the transcripts. In the second phase, TJO and RGS independently fragmented and coded the transcripts inductively. They coded the first interviews independently and generated an initial list of codes. Memos about contradictions or additional questions were added. The initial list of codes and memos was discussed and if necessary, some alterations were made. In the third phase, the codes were sorted into initial themes. Patterns among the codes were identified by constant comparison. Researcher LZ joined the analysis process at this stage. The themes were visually represented in a thematic map. In the fourth phase, the themes were reviewed, discussed and revised with a senior researcher (MHD). In the fifth phase, the themes were refined and used as a framework for the remaining interviews. If new patterns and themes emerged, these were added to the existing map. MAXQDA version 2021 (VERBI Software 2021) was used to organize all data [15]. In the last phase, narratives were written together with data extracts, and contextualized using literature. The interviews and analysis were conducted in Dutch. Only quotations used in the article were translated from Dutch to English by the researchers (TJO, RGS and LZ) and checked by the research team.

### Rigor

To enhance credibility, summaries of the transcripts were sent to the participant it concerned. Participants were asked whether the summary was accurate, and if they would like to clarify or rectify their responses [16, 17]. Participants agreed or made some changes or suggestions. The topic list was reviewed by the researchers who conducted the interviews after each interview and the topic list was adjusted, if necessary (see Appendix A). Baseline characteristics are provided to describe the context of each participant (see Table 1). Lastly, to ensure confirmability, the researchers (TJO, RGS, LZ) collaboratively developed themes based on the interviews. These were then discussed with MHD (peer debriefing).

### Ethical considerations

The study protocol was approved by the Medical Ethics Review Committee of MMC. Study procedures followed were in accordance with the Declaration of Helsinki. The study aim was explained to participants prior to the interview. Informed consent from all participants was obtained. Confidentiality was assured using restricted access to the data and de-identification of the transcripts. Interview data was stored on a secured online research drive.

## Results

### Patient characteristics

Fourteen participants, aged 30–59, were interviewed. The majority reported experiencing symptoms of HMB for more than five years. Additionally, participants received various treatments for their symptoms. Most participants were treated with LNG-IUD or COC, and fewer with an EA or a hysterectomy. See Table 1 for a complete overview of the participants’ baseline characteristics.

**Table 1:** Baseline characteristics of the participants (*n* = 14)

	Age group (years)	Nationality	Highest level of education	Duration of symptoms (years)	Symptoms reported	Received treatment(s)
1	40–44	Hungarian/ Dutch	University	5–10	HMB, Fatigue	LNG-IUD
2	55–59	Dutch	University	> 15	HMB	COC, LNG-IUD, EA
3	50–54	NA	NA	> 15	HMB, Met	COC, EA, OT
4	45–49	Dutch	University	5–10	HMB, Dys	COC, LNG-IUD, EA + LNG-IUD
5	55–59	Dutch	HPE	< 5	HMB	COC, LNG-IUD
6	50–54	Dutch	HPE	< 5	HMB, Dys	COC, LNG-IUD, OT
7	45–49	Turkish	SVE	> 15	HMB, PMS, Dys, Fatigue	COC, LNG-IUD
8	55–59	Dutch	University	10–15	HMB, Met, Dys, PMS	COC
9	35–39	Dutch	SVE	5–10	HMB, PMS	OT
10	30–34	Dutch	SVE	5–10	HMB, Met, PMS	COC, OT
11	50–54	Dutch	NA	10–15	HMB, Fatigue	COC
12	50–54	Dutch	University	> 15	HMB	COC
13	50–54	Dutch	University	> 15	HMB	COC, LNG-IUD, OT, HE
14	40–44	Dutch	HPE	< 5	HMB	COC, OT, HE

LNG-IUD = Levonorgestrel releasing intra-uterine device, COC = Combined Oral Contraceptive, EA = Endometrial ablation, HE = Hysterectomy, OT = Other treatments such as (contraceptive injection, Implanon, Hormone therapy, NuvaRing, Tranexamic acid), HMB = Heavy Menstrual Bleeding, Dys = dysmenorrhea, PMS = pre-menstrual syndrome, Met = Metrorrhagia, NA = No Available data, HPE = Higher professional education, SVE = senior Secondary Vocational Education

Based on analysis of the interviews, three main themes and eleven subthemes emerged.

### Theme 1: considerations in taking the (next) step to seek help

Despite the significant impact of severe bleeding on their daily life, participants find it difficult to seek help for HMB. Most participants preferred a treatment without hormones. Some participants expressed they hoped for a natural solution, like menopause.

#### Impact on daily life stimulates seeking help (again)

Participants described their menstruation as heavy, illustrating this by examples of the amount, unexpectedness or duration of blood loss, and the impact on their daily lives.

*“Sometimes I was bleeding for 6–7 weeks in a row and then I wasn’t for 3 months*,* and then I was again for a day. I could not make any sense of it” (Participant 3)*.

Participants mentioned that staining and leaking from HMB had major impact on their daily lives. Several participants explained how heavy bleeding during the night disrupted their sleep, requiring them to wake up every few hours to change menstrual products. Moreover, some participants expressed feeling ‘dirty’, or ‘unfresh’ due to HMB.

*“Judo is a full contact sport*,* and if you don’t feel fresh*,* it is very difficult to be close to others. So that really bothered me for a very long time” (Participant 14)*.

Many participants mentioned adopting precautionary measures to manage HMB. For example, participants used different menstrual products simultaneously, such as a tampon and a sanitary pad. Others explained the need to bring an extra pair of clothes to work or when traveling outside.

*“I’ve had to uh*,* uh*,* change pads and tampons every hour*,* so- And then uh*,* I always had extra clothes with me. So*,* so um*,* I just couldn’t do anything. I couldn’t leave {the house}*,* so to speak. I couldn’t even work anymore. Yes*,* I was there*,* but um*,* with an eh*,* with a pack of sanitary pads and tampons in front of me.” (Participant 13)*.

Several participants were motivated to seek medical help due to the significant impact of HMB on their daily lives. Many participants described reaching a personal threshold or experiencing a change in symptoms, leading them to visit their GP for help.

*“Even at night I had a tampon in and I had a sanitary pad and even then*,* my whole bed was destroyed. I just had to get new mattresses*,* because you just leak through. And then you’re like now something has to be done.” (Participant 2)*.

#### Seeking help is hindered by embarrassment

Few participants found it embarrassing to go to the GP to talk about their menstrual complaints and expose their bodies. They found it very unpleasant to undergo vaginal examinations, especially when they are menstruating. This is a reason for participants to delay seeking help.

*“But I always think that it is the most um*,* well I really go to a doctor a lot*,* but um*,* this kind of appointments*,* I find*,* I find it embarrassing. I don’t know. It makes me feel uncomfortable as a woman.” (Participant 8)*.

#### Menopause as a solution

Other participants hoped for menopause as a natural solution. Many participants dealt with symptoms of HMB for many years. Therefore, waiting for menopause was considered as an alternative to the non-invasive or surgical treatments. Some participants said that they would rather wait a few more years until they reach menopause than starting another treatment.

*“I think it {a hysterectomy} is very rigorous. And that’s a pretty big surgery*,* um*,* with the necessary- Yes*,* longer recovery also um*,* um*,* yes*,* I’m also with age considering- I think- I’m like*,* yes*,* suppose the menopause is just- Yes*,* coming up*,* then it’s also something of*,* then I’d rather wait for that.” (Participant 9)*.

### Theme 2: various sources of information can contribute, confuse or frighten decision-making process

Different sources of information contributed to a participant’s knowledge of different treatment options and therefore their decision-making. These sources included one’s own experiences, experiences or opinions of others, online information, and information and opinions provided by the physician. Sometimes knowledge also caused fear or uncertainty for a particular treatment or result.

#### Information provided by physicians

Information received by the participants varied enormously. Some participants were well prepared for the risks and benefits of a treatment, while others were completely overwhelmed by post-operative complications or side effects. Most participants received a leaflet with information about a treatment or were advised to read information on a website provided by the gynaecologist.

*“I was just given a leaflet*,* and it just says how the treatment works and not what might happen afterwards. Yes*,* they tell you that you could have less blood loss and that you are going to have that novasure treatment {endometrial ablation}. […]. I just read the leaflet about how or what the treatment itself entails.” (Participant 3)*.

An option grid provides information on possible risks and complications of all treatment options for HMB. Many of the participants did not recognize the option grid or were not aware of all the possible treatment options. One participant mentioned that the use of an option grid would be useful if she did not know all the possible treatment options.

*“So if I didn’t work in healthcare*,* I*,* I wouldn’t*,* I wouldn’t know those treatments*,* they wouldn’t have said anything to me*,* so eh- At least*,* an IUD yes*,* but those medications without hormones and a NovaSure treatment*,* that wouldn’t really have said anything to me*,* I think. So I*,* I would like this*,* this {option grid}.” (Participant 13)*.

Specifically, most participants reported being unprepared for the abdominal pain after EA.

*“Well*,* I had a really bad stomachache 7 to 14 days {after treatment}. When I pointed that out*,* they {the physicians} said: ‘yes*,* that’s part of it’. This cannot be part of it. I believe I take 600 mg of ibuprofen*,* three to four times a day. Then I thought*,* this is not normal.” (Participant 2)*.

#### Social environment steers and confuses decision-making process

In the interviewee’s social environment, diverse opinions and experiences regarding treatments for HMB played a role in the decision-making process. Some participants reported negative experiences from their social environment about certain treatments. These experiences contributed to a patient’s treatment decision.

*“No*,* because I have two friends who had IUDs and they got pregnant with twins. So that didn’t work out either. And they weren’t yet*,* they did not have a desire to have children*,* (…) So you always have to weigh up what you can and can’t do and the experiences of others. And that’s what you actually do it with.” (Participant 10)*.

In addition, there were numerous stories about adhesions and pain after EA that contributed to participant’s treatment decision.

*“Because I read that some people did have pain symptoms from that that {endometrium ablation}. And that I thought*,* yes*,* always stomach pain*,* um*,* that really held me back.” (Participant 12).*

*“But that there could also be adhesions. So that was actually for me- Anyway also from my acquaintance where it didn’t help either*,* but also those adhesions and that you then also had to go back to the gynecologist continuously*,* that actually made me decide a little to refrain from that. (Participant 7).*

#### Need for self-acquired information

Another source of knowledge for participants was self-acquired information. Many participants explained the necessity of searching stories, experiences online and more information about the procedures. While some information contained negative experiences about treatments gone wrong, there were also stories about the positive effects of treatments. However, many participants emphasized that their primary goal to look up information was to adequately prepare themselves.

*“Certainly not because not all the stories you read are equally positive. Hey*,* it’s*,* it’s also*,* there are some horror stories*,* I might be too down-to-earth for that. Of course*,* it depends on each person and what is your pain threshold. And what do you think is a lot and what kind of things do you mind? So*,* you have to filter that. And there were very few positive stories*,* about how well things are going. So you don’t find those either.” (Participant 4)*.

One participant searched online for videos and pictures of various treatment procedures.

#### Past experiences influence decision-making

One’s own previous experience with a particular treatment also plays a significant role in the decision-making process. Many participants mentioned a negative experience with the side effects of hormonal treatment. These side effects included mental health issues (mood swings and depression), making them reluctant to choose treatments containing hormones.

Participants mentioned that taking hormones did not feel natural. They also expressed that hormonal treatments did not always reduce blood loss or premenstrual symptoms.

*“Nausea*,* vomiting*,* headaches. Yes*,* headache and- Look*,* swollen breasts and belly you always have. But it was like when I took the pill that it got much worse.” (Participant 7)*.

However, other participants had good experiences with hormones (COC or LNG-IUD), which made them feel positive about those treatments and ultimately made the choice of treatment easy. One participant mentioned when discussing the endometrial ablation and the endometrial ablation combined with the LNG-IUD, that she would prefer the latter option based on previous experiences with the LNG-IUD.

#### Expectations and fears prior to treatment steer the decision

While some participants considered treatment as a solution to their complaints, others perceived it as an obstacle. Participants mentioned fear of complications or worsening of the symptoms as a consideration to reject a specific treatment.

“*I mean*,* if you go for a treatment*,* then*,* of course you want your problem to be solved in the end*,* but also you don’t want a new problem to arise. Or another problem or*,* or the same problem in*,* in- Yes*,* that*,* you don’t want that. You want your problem to be solved in the end.” (Participant 9)*.

The potential side effects of treatment on their daily life were also a consideration. For example, abdominal pain after undergoing an EA and an inability to continue with daily activities were considered as disadvantages of EA. Other participants did not prefer a hysterectomy. This treatment was seen as invasive and painful, and it required an extended recovery time.

*“Um*,* well the pain um*,* I was um*,* very afraid of it anyway. And I had heard stories about other people who had been in a lot of pain. So I was*,* I thought*,* very exciting. And the period afterwards of eh*,* I’m quite a busy bee. And then the six weeks of doing nothing and*,* eh*, *basically not being allowed to lift a carton of milk*,* I really dreaded that.” (Participant 14)*.

Participants who underwent a hysterectomy expressed that taking a short break from daily activities due to the surgical procedure was perceived as a minor setback opposed to the disadvantages of HMB. For example, the following participant already underwent three treatments before a hysterectomy and she described her decision leading to the hysterectomy as a tough journey:

“*Um*,* so those advantages did far outweigh the disadvantages for me. The disadvantages are that you just have a long recovery*,* it’s quite an operation and I was a bit worried about that. But um*,* it’s also been a tough journey in the end*,* because it was a tough ok*,* but um*,* yes*,* it’s the best decision I could have made*,* I think.” (Participant 13)*.

At first this participant was disappointed with what she could do during recovery, but when she was fully recovered, she was satisfied with the end result.

*“During recovery um*,* yes*,* at the time I was disappointed with what I could actually do*,* so to speak. I couldn’t do anything. Um*,* and I really had to take a lot of rest and when I was fully recovered*,* yes*,* that was absolutely super*,* no more trouble from anything and eh*,* yes*,* ideal. I should have done that much earlier.” (Participant 13)*.

The majority of the participants suffered from HMB for several years and expressed the desire for a mild or regular menstruation, or no menstruation at all. They emphasized that the burden of their symptoms affected their daily lives. Anticipated treatment effects such as reduced blood loss and increased control over their lives were mentioned as factors influencing their treatment decisions.

When asking their initial preferences for treatment, some participants indicated they preferred the least invasive treatment, such as hormonal contraception. One participant mentioned preferring an LNG-IUD opposed to COC, since it does not require taking a pill every day.

*“I don’t want to deal with it. I just want Mirena in and be done. Hopefully then it will be inserted properly. And they’re just going to monitor it really well this time*,* every so often they’re going to check to see if it’s still in place. So you know*,* then you don’t have to worry about it anymore”. (Participant 7)*

In contrast, some participants considered hormonal contraception (taking a pill daily) as a simple treatment.

Many participants favored a hysterectomy, viewing it as a definitive solution. They mentioned benefits such as ‘getting rid of all the symptoms’ or no more blood loss. Some of the participants had no desire for additional children and considered their uterus as unnecessary. One participant mentioned that if a hysterectomy would eliminate premenstrual symptoms, she would prefer that.

“*Coming from me*,* I would have said: take out the whole uterus and be done with it. I would have said the same thing 10 years ago*,* gee*,* I don’t mind if it’s taken out.“ (Participant 3)*.

### Theme 3: A physician’s understanding and a relationship of trust are needed to guide the decision-making process

A physician’s attitude, the feeling of being heard and room to express one’s own choice during the decision-making process affected the experience of the participants. Most participants highly valued the opinion of their physician and some followed their advice.

#### The importance of the feeling of being heard

Participants emphasized the importance of the relationship with their treating physician in their decision-making. To them, being heard and taken seriously during consultation was important for their appraisal of the relationship.

*“When the internist took me seriously about the anemia*,* that really made me feel supported. Because earlier*,* I have also been to an internist and they were like*,* yeah*,* well*,* it’s part of it*,* end of story.” (Participant 12)*.

Although some participants were appreciative of the relationship, others did not feel heard by their physician. Some participants mentioned that the physician did not notice their symptoms of HMB at first. Others mentioned that their physician did not take their pain seriously, which made some participants feel like their complaints were “trivial”. All these factors led to a feeling of not being heard by a physician.

*“Well*,* I think I had a really bad stomachache 7 to 14 days {after treatment}. And if you indicate that and they {physician} say: ‘yes*,* that’s part of it’. Then I think: this is not part of it*,* I believe I took ibuprofen 600 mg and then three to four a day. Then I think: I don’t think this is normal. There was something wrong with that. Well*,* that was not picked up. I regret that.” (Participant 2)*.

#### Room for expressing one’s own choice next to the physician’s opinion is important

Participants mentioned the importance of expressing one’s own treatment preference. They expressed that an open relationship with their physician made it easier to express their wishes. Participants expressed that they appreciated being given the time to consider different treatments and make a shared decision. In contrast, in a relationship in which a physician is very directive, little space is left for a patient’s own choice.

*“Well*,* I actually wanted to go for Novasure {endometrial ablation} then*,* but then they thought I was too young for that. Then I was about forty and then they thought I was too young for that*,* while*,* I knew myself that I had no desire to have children*,* eh*,* I had no partner at that time” (Participant 11)*.

Participants often expressed that their treating physician strongly suggested which treatment the participant should undergo. Consequently, not all treatment options were presented to the patient. In particular, the option to perform a hysterectomy was often not discussed or was very quickly discarded for various reasons.

*“Yes*,* too young and all that*,* and we can always try other treatments. I say*,* yes other treatments*,* I don’t see the point. If someone suffers so much*,* and not just for a moment but for a number of years. I don’t want to have children*,* never have and never will. And it’s the patient’s wish*,* “Oh*,* I don’t mind having my uterus removed. I don’t see the point in it not being allowed or being possible”. Because then I think*,* as a patient*,* I am actually being hindered in my choice*,* because they have already made a choice for me not to do it. While they do mention it as an option to be able to do it*,* ultimately”. (Participant 3)*

#### Trusting the physician matters

Participants also mentioned that trust in the relationship with the physician is key. Some participants fully trusted their physicians and let them decide.

*“No*,* I didn’t have eh*,* no I didn’t have myself oh*,* I think*,* if I go to the gynecologist*,* he must know what is best […] And burning {ablation} thought my gynecologist*,* he’s not in favor of that. So that was already measuring with him of we’re not going to start that*,* because I don’t like it*,* because that’s more um*,* causing problems than it is solving. That was his own opinion*,* so I think*,* then I follow his opinion.” (Participant 10)*.

Nevertheless, other participants mentioned distrusting their physician or their competence. Reasons to distrust a physician included: inattentiveness to symptoms, rumors about specific physicians, or instances where a general practitioner (GP) refused to refer a patient to a gynecologist. For some participants, this resulted in avoiding appointments with their physician.

*“Well*,* he checked*,* but yes*,* he thought I was squeamish. Well*,* okay. Yes*,* sorry*,* but the treatment itself*,* I found*,* compared to the pain after. So*,* just to put it in perspective. So in your eyes I might be squeamish*,* but yes*,* you don’t know the background*,* I think at that moment. […] If I had known that he was the gynecologist on duty*,* I would not have gone. I say very honestly*,* in retrospect. Because I heard several stories about him”. (Participant 4)*

## Discussion

The aim of this study was to gain insights in the experienced impact of HMB and the motives and considerations of women during the decision-making process regarding treatment options for HMB. The three main themes that emerged are “Considerations in taking the (next) step to seek help” (Theme 1), “Various sources of information can contribute, confuse or frighten decision-making process” (Theme 2) and “A physician’s understanding and a relationship of trust are needed to guide the decision-making process” (Theme 3).

The burden of HMB on daily life was an important reason to seek for help, but differed enormously between the participants. This is in agreement with Chapple et al. (1999) who found that symptoms which “started to disrupt their lives were commonly the trigger to seek medical help and differ between women” [18]. The process of seeking help consists of different steps. Initially, the perception of menstrual bleeding as ‘abnormal’ must be acknowledged, followed by the decision to determine the legitimacy of seeking medical help. The interviews revealed that some participants had a negative connotation towards hormone-containing treatments, mainly due to the perception of administered hormones not being natural. The study of van den Brink et al. confirms this finding, as further explained below [19].

Women’s knowledge about the different treatment options comes from a variety of sources, such as the internet, their own experiences, others’ experiences and opinions, and the opinions of their physicians. In the study of Vuorma et al. (2003), approximately one in three women (17–34%) felt like they were not sufficiently informed by the physicians on the benefits and complications of alternative treatment options for HMB. Preferences regarding treatment were most strongly associated with women’s pre-visit preferences [20, 21]. Many participants considered treatment for HMB as an obstacle, as they feared side effects, complications and disruption of daily life. Participants were also afraid that treatment would not provide a definite solution or might worsen their symptoms. Therefore, many participants favored the least invasive treatment. This finding is also in line with the results of van den Brink et al. (2018) [19]. They studied women’s preferences for treatment of HMB and concluded that the presence of hormones, the (ire)reversibility of treatment and effect on irregular bleeding were important in making a treatment decision. In our study, participants mentioned the presence of hormones as a factor influencing their decision to not choose a specific treatment. Nevertheless, the study of van Den Brink does not elaborate on the specific motivations of women during the decision-making process. However, it is important to note that participants differed in their preferences. While some participants preferred non-invasive treatments, others favored a hysterectomy, as it offers a definite solution for their complaints.

The experienced relationship with a physician played a major role in the participant’s treatment decision-making. Participants expressed that the feeling of being heard, trust in the physician and being able to express one’s own choice were important in the appreciation of the relationship. Experiences of participants varied, some valued the relationship with their physician, while others felt distrust towards their physician. The opinion of physicians seems to dominate the decision-making process. Eising et al. (2018) explored key factors for successful support in patients with Von Willebrand disease, faced with HMB [22]. Their research found that a precondition for support is a good relationship with a physician: a trusting relationship, where information can be shared. The findings in this study are in line with findings from Eising et al. (2018). According to the research of Skea (2004), 75% of the women who underwent a hysterectomy for HMB preferred to make the decision together with their physician [23]. 4% of women preferred the physician to make the decision, while 2% wished to make their own decision.

The desired outcome of the treatment varied among participants, ranging from achieving a mild menstruation through a simple treatment to seeking a definitive solution by undergoing an invasive treatment such as a hysterectomy. The participants had varying expectations regarding the treatment, including considerations of its invasiveness or effectiveness. These expectations steered the final treatment decision. Kennedy et al. (2003) conducted a randomized controlled trial to evaluate the effect of structured preference elicitation interviews during the decision-making process for treatment of HMB [24]. The first group received a video explaining the different treatment options, while the second group also met with a research nurse to discuss different treatment options. Concluding, women who got a chance to elaborate their preferences, underwent second invasive treatment (hysterectomy) less often after two years of follow-up. Also, satisfaction rates were higher and health care costs were lower in the group of women who were seen by the research nurse. This is in line with the results of our study. This highlights the importance of understanding woman’s motivations and considerations when deciding on a treatment.

A helpful tool to counsel the advantages and disadvantages of the various treatment options in the consulting room is the use of an option grid, commonly used in the Netherlands. This grid briefly explains the success rates of the various HMB treatments as mentioned above, as well as the benefits and complications. In this study, we sent an option grid prior to the interview, which we used as an overview of the possible treatments offered in the Netherlands. We did not study the usefulness of the option grid, but we asked if the participants recognized the grid. More important, we asked if they recognized all the treatment options. It was noticeable that most participants were not aware of all the treatment options available for HMB. A helpful way to explain all the treatment options during consultation is to use an option grid.

### Strengths and limitations

A strength of this study is that by using in-depth interview methods we were able to foster a safe environment to gain the participants’ trust to share their experiences. Another strength of this study is that we interviewed women who had experienced HMB multiple years and were therefore at different stages of the disease with a variety of treatment experiences. We aimed to ensure transferability by providing baseline characteristics of each participant. Additionally, the topic list was discussed by two researchers after each interview and adapted after five interviews. To ensure dependability, we collected data until no new themes emerged. After eight interviews, no new main themes emerged. In addition, we analyzed the data iteratively and we refined the themes if necessary. Finally, to ensure credibility, participants received a summary of the results and were asked feedback on the interpretation of the results. All participants responded and seven participants did not suggest any changes or clarifications.

Our study was limited to the experiences and considerations of women who received treatment of HMB. Experiences of women who have never sought medical consultation or consulted a physician but refrained from treatment were not included. Since we recruited participants through questionnaires and flyers, it is possible that self-selection bias occurred. It is possible that we only interviewed participants who were comfortable with sharing their stories, leading to a bias in the data [25]. It is also possible that the members of the Netherlands Patients Federation who participated in this study were more assertive than the average patient. Amongst the participants, there is diversity in terms of age, education level, years of experience with HMB and type of treatment. Unfortunately, there is little variation in nationality. As a result, topics such as culture and religion and whether these factors play a role in considerations for treatments are not included. The data was retrospectively collected, which may have increased the possibility of recall bias. Women who consulted a physician five years prior to the interview were eligible for our study. Recall bias was minimized by recruiting women through the patient clinic of MMC who were in the midst of their treatment. Motives and considerations regarding the received treatment may be influenced by the effectiveness of the received treatment, the moment in the patient journey and previous experiences of treatments for HMB. Also, the interviews were conducted by two researchers, of which one is a medical doctor. This may have impacted openness of the interviewee due to perceived formality. In addition, the doctor’s presence may have introduced response bias. To address this, we implemented rapport building and engaged in reflexive practices. Lastly, the interviews were conducted in Dutch therefore the quotes were translated into English by the research team. As a result, the emotion of the quotes could get lost in the translation.

**Recommendations** Our results suggest that a patient’s treatment decision is influenced by the attitude and communication of their physician. The findings of this study allow for the formulation of a few recommendations. Most significantly, it is critical to foster an environment of open communication during consultation so that the patient feels at ease with the physician. Patient-centered communication is a helpful communication tool to use during consultation [11]. It acknowledges the entire person, their personality, life history, and social structure in order to develop a shared understanding of the problem, treatment goals, and barriers to those goals. This allows us to gain an understanding of the motivations for a certain belief, experience, or knowledge from the perspective of women. It is also important to consider that shame and discomfort may play a role in the decision to consult a physician.

Participants of this study particularly valued being informed about all possible treatment options for HMB, including the advantages and disadvantages of each treatment. The option grid can be a helpful tool to use during the decision-making process.

In future research, we recommend a prospective study with a long follow-up, following women from the onset of their patient journey to investigate all possible factors influencing treatment decisions.

## Conclusion

This study provides insights into patients’ motives and considerations during decision-making for treatments of HMB, but also in the long and confusing journey some participants experienced. The first obstacle to overcome for women with HMB is to decide that help is needed (again). Main considerations in the decision-making process include obtained information and experience, relationship with the physician, influence of the social environment, pre-visit expectations/desires, fear of treatment complications and uncertainty of treatment effect. It is the responsibility of the physician to create a trusting and open atmosphere during consultation. Patient-centered communication is helpful to share knowledge, and gain insight into patients’ hopes, fears and worries. Additionally, it is essential to offer the patient comprehensive information to support informed decision-making.

## Data Availability

The datasets used and analysed during the current study are available from the corresponding author on reasonable request.

## Abbreviations

HMB: Heavy menstrual bleeding
LNG-IUD: Levonorgestrel-releasing intra-uterine device
EA: Endometrial ablation
NPF: Netherlands Patient Federation (Dutch patient federation). See also: https://www.patientenfederatie.nl/netherlands-patients-federation
COC: Combined oral contraceptive
